# Unpacking the indirect association between problematic short-form video use and appearance anxiety

**DOI:** 10.1186/s40337-026-01549-2

**Published:** 2026-02-14

**Authors:** Xiangling Hou, Mu He, Ziying Han, Xu Liu, Suying Li, Xianglian Hou, Dan Li

**Affiliations:** 1 School of Educational Sciences, Chongqing Key Laboratory of Psychological Diagnosis and Education Technology for Children with Special Needs, Chongqing Normal University, Chongqing, 401331 China; 2 School of Foreign Languages and Literatures, Chongqing Normal University, Chongqing, 401331 China; 3 Positive Psychological Experience Center, Chengdu Vocational & Technical College of Industry, Chengdu, 610218 China; 4 Jinwutang Town School, Shaoyang, 422608 China; 5 College Student Mental Health Education and Consultation Center, Hainan Medical University, No. 3 Xueyuan Road, Longhua District, Haikou, 571199 Hainan The People’s Republic of China

**Keywords:** Problematic short-form video use, Appearance anxiety, Self-esteem, Boredom proneness, Young adults

## Abstract

**Background:**

Appearance anxiety has become a growing public health concern among young adults; however, its psychological correlates remain inadequately explored, particularly within the realm of digital media engagement. This study examined the associations between problematic short-form video use and appearance anxiety, and whether self-esteem and boredom proneness accounted for part of this association.

**Methods:**

A sample of 411 Chinese college students completed a battery of standardized scales assessing problematic short-form video use, self-esteem, boredom proneness, and appearance anxiety.

**Results:**

Problematic short-form video use showed a large positive association with appearance anxiety. This association was partly explained by self-esteem and boredom proneness, both independently and sequentially, indicating significant indirect paths via lower self-esteem and higher boredom proneness.

**Conclusions:**

These findings indicate that self-esteem and boredom proneness are important correlates linking problematic short-form video use and appearance anxiety among young adults. Although the cross-sectional design precludes causal inferences, the results point to the potential value of enhancing self-esteem and helping young people manage boredom may be promising avenues for future intervention efforts.

## Abstract (plain language summary)

Anxiety about one’s physical appearance is a noteworthy phenomenon in young adults in the digital age. Problematic short-form video use has been linked to appearance anxiety, and self-esteem and boredom proneness may play important roles in this association. The present study revealed that individuals with higher levels of problematic short-form video use were more likely to report appearance anxiety. This association could be partly explained by lower self-esteem and higher boredom proneness, both independently and sequentially. In other words, young adults with more problematic short-form video use tended to have lower self-esteem and to feel bored more easily, which in turn associated with greater anxiety about their appearance. Although these results cannot infer cause and effect, they suggest that enhancing self-esteem and managing boredom may be promising avenues for helping young adults cope with appearance anxiety in the context of short-form video use.

## Introduction

Global reports suggest that appearance-related concerns are increasingly salient among young adults. For example, the International Society of Aesthetic Plastic Surgery (ISAPS) Global Survey [1] has documented a continuing rise in aesthetic surgical and non-surgical procedures worldwide, reflecting growing demand for appearance-related services and elevating levels of appearance anxiety. Against this backdrop, appearance anxiety, characterized by excessive worry about one’s looks, often motivated by perceived social standards and fear of negative evaluation [2], has attracted increasing scholarly attention. This condition has been linked to a variety of detrimental outcomes, such as diminished quality of life [3], impaired physical performance [4], and disordered eating behaviors [5]. These consequences highlight the critical need to identify and examine underlying contributing factors.

One potential contributing factor is problematic short-form video use. Short-form video platforms can be viewed as a subtype of social media, yet they incorporate distinctive and highly engaging design features (e.g., short, rapidly rewarding clips) that may promote more intensive and repetitive viewing than many traditional social media platforms. They also contain a high density of visually oriented, appearance-salient content (e.g., curated selfies and beauty filters) alongside immediate social-evaluative cues (e.g., likes and comments). As such, short-form video use may be particularly relevant for understanding appearance-related anxiety. Platforms like Instagram and TikTok can intensify exposure to curated and idealized representations of appearance, potentially reinforcing narrow and often unattained beauty standards [6, 7]. Grounded in social comparison theory [8], which posits that individuals are driven by an innate tendency to evaluate themselves through comparisons with others to reduce uncertainty and shape self-concept, these image-centric environments may foster upward social comparisons [9]. Consistent with these views, emerging empirical evidence indicates a positive correlation between short-video social media exposure and appearance anxiety [10].

Nevertheless, the relationship between problematic short-form video use and appearance anxiety remains underexplored. In particular, the strength of this association and the potential indirect roles of personality traits, whether adaptive, such as self-esteem [11], or maladaptive, such as boredom proneness [12], have not yet been empirically established. Importantly, appearance anxiety is not only a salient psychological concern in its own right but has also been linked to a range of adverse outcomes, including disordered eating behaviors [5]. From this perspective, clarifying how problematic short-form video use relates to appearance anxiety may have broader implications for understanding vulnerability to eating-related problems. Specifically, frequent exposure to highly curated and idealized body-related content on short-form video platforms may intensify appearance-related self-monitoring and social comparison, which have been associated with body dissatisfaction and disordered eating risk [6, 7]. Understanding the pathways between problematic short-form video use and appearance anxiety may provide valuable insights for prevention and intervention of appearance anxiety and eating disorder behaviors.

Therefore, the current study aims to explore the indirect effects of self-esteem and boredom proneness in the association between problematic short-form video use and appearance anxiety among young adults.

### Problematic short-form video use and appearance anxiety

Problematic short-form video use is defined as a pattern of compulsive engagement with short-form video platforms, marked by intense and persistent cravings to watch videos and a diminished capacity to regulate usage, which can interfere with daily functioning and overall well-being [13]. A growing body of research indicates that this problematic behavior is associated with adverse consequences for both physical and psychological health [14, 15].

Grounded in objectification theory [16], sociocultural environments are proposed to encourage individuals to adopt an observer’s perspective on their own bodies, which may be associated with heightened body surveillance and self-scrutiny focused on physical appearance. In this framework, the appearance-focused content and evaluative cues that are common on short-form video platforms may reinforce objectifying appraisals and social comparison, potentially contributing to higher levels of appearance anxiety. Empirical work has demonstrated a consistent positive association between social media addiction and appearance anxiety [17, 18], with recent studies specifically linking short-form video exposure to increased anxiety surrounding appearance [10]. This effect is particularly pronounced when users engage with content featuring beauty filters or body-focused narratives, which have been shown to provoke heightened appearance-related distress [7, 19]. Furthermore, algorithm-driven content delivery systems on these platforms may perpetuate a cycle of exposure by continuously curating idealized and often unattainable appearance standards, thereby intensifying negative self-perceptions. Notably, adolescents and young adults may be especially vulnerable to these influences. During this critical developmental window marked by identity formation and body image development [20], yet characterized by still-maturing cognitive and emotional regulatory capacities [21], youths may be particularly susceptible to external visual and social cues. It is therefore plausible that problematic short-form video use is positively associated with appearance anxiety in young adults.

### The indirect effects of self-esteem and boredom proneness

Self-esteem refers to an individual’s overall subjective evaluation of their own worth and capabilities [22]. Higher levels of self-esteem serve as a protective factor, positively correlating with enhanced life satisfaction and healthier social relationships [23, 24]. Conversely, low self-esteem is associated with an increased risk of anxiety, depressive symptoms [25], and dating violence [26].

Rooted in social comparison theory [8], self-esteem can be understood as partly shaped by individuals’ self-evaluations relative to others, such that frequent upward comparisons may undermine perceived self-worth. From this perspective, self-esteem may play a critical role in the association between problematic short-form video use and appearance anxiety. On short-form video platforms, appearance-focused content (e.g., beauty filters and cosmetic surgery showcases) is commonly encountered [9]. Exposure to such content may reinforce narrowly defined and often unattainable beauty ideals and encourage upward appearance-related social comparisons [9]. Chronic exposure to these idealized portrayals may undermine an individual’s self-image and sense of self-efficacy, and ultimately relate to reduced self-esteem [27]. These associations may be particularly salient during adolescence and emerging adulthood (relative to older adulthood), given that body-image concerns are typically more prominent in younger populations and that image-focused social media platforms are especially popular among young people, potentially increasing exposure to appearance-related feedback and comparison opportunities [28]. A body of empirical research corroborates this perspective, having consistently identified a negative association between social media addiction and self-esteem [29–31].

In addition, self-esteem is considered a significant internal psychological resource that helps individuals regulate emotional responses to appearance-related evaluation. Those with higher levels of self-esteem tend to possess a more resilient and integrated self-concept, rendering them less vulnerable to adverse social comparisons and societal beauty standards [32]. Consequently, such individuals are better equipped to maintain a positive and stable self-perception even when frequently exposed to idealized portrayals on social media [27, 33]. Empirical studies have consistently demonstrated an inverse relationship between self-esteem and appearance anxiety [11, 34, 35], supporting the proposition that self-esteem serves as a significant indirect factor in the relationship between problematic short-form video use and appearance anxiety.

Boredom proneness may also operate as an indirect factor in the association between problematic short-form video use and appearance anxiety. Although transient boredom can sometimes serve adaptive functions (e.g., motivating exploration or creativity), boredom proneness refers to a stable dispositional tendency to experience boredom frequently and across situations, especially in low-stimulation or repetitive situations, and has been conceptualized as a risk-related trait rather than a momentary state [36]. Consistent with this view, boredom proneness has been linked to maladaptive psychological outcomes, including lower life satisfaction [37], higher depression and anxiety [38], and poorer emotion regulation [39].

Although short-form video platforms are engineered to deliver constant sensory stimulation and engaging content, excessive consumption may paradoxically be associated with increased, rather than reduced, boredom [40]. This counterintuitive effect was supported by a longitudinal investigation of 699 college freshmen, which found that problematic online video viewing positively correlated with subsequent boredom proneness [12]. From the standpoint of self-regulation theory [41], which posits that individuals modulate their behavior, emotions, and cognitions in pursuit of goal-directed actions, boredom and under-stimulation can disrupt these processes. When experiencing boredom, individuals often develop negative attitudes toward tasks and exhibit impaired self-regulatory capacity [42]. Such regulatory deficits may lead to disengagement, passive media consumption, and heightened susceptibility to negative emotional states, including anxiety [43].

Furthermore, the algorithmically curated repetition of content, often emphasizing idealized beauty standards and aspirational lifestyles, can induce frequent upward social comparisons. These comparisons, particularly under conditions of reduced cognitive engagement and elevated boredom proneness, may increase susceptibility to maladaptive cognitive patterns and intensify appearance-related concerns [44]. Young adults may be especially vulnerable to the synergistic effects of immersive short-video environments and individual predispositions toward boredom, thereby elevating their risk of developing appearance anxiety. It is thus plausible that boredom proneness plays a salient indirect role in the relationship between problematic short-form video use and appearance anxiety.

Growing evidence indicates that self-esteem may act as an antecedent variable that influences boredom proneness. Individuals with lower self-esteem may be less likely to experience a stable sense of competence and personal value, which can weaken intrinsic motivation and reduce engagement in purposeful activities, both of which are established antecedents of heightened boredom susceptibility [45, 46]. This proposition is empirically supported by studies documenting a significant negative association between self-esteem and boredom proneness [47]. Therefore, it is plausible to hypothesize that self-esteem and boredom proneness together form a sequential pathway linking problematic short-form video use to appearance anxiety.

### The present study

**Fig. 1:**
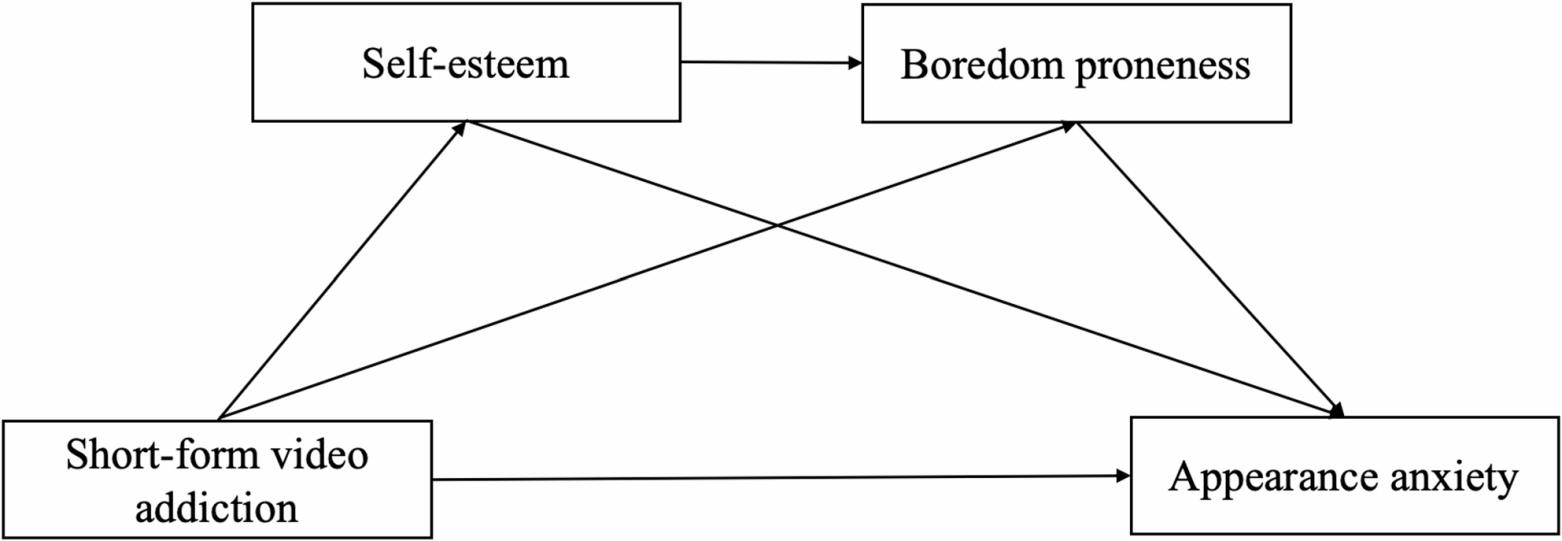
The conceptual model

This study sought to investigate the relationship between problematic short-form video use and appearance anxiety among young adults, and the indirect effects of self-esteem and boredom proneness (see Fig. 1 below). The following hypotheses were proposed:

#### H1

Problematic short-form video use would be positively associated with appearance anxiety;

#### H2

 Self-esteem would play an indirect role in the relationship between problematic short-form video use and appearance anxiety;

#### H3

 Boredom proneness would play an indirect role in the relationship between problematic short-form video use and appearance anxiety;

#### H4

 Self-esteem and boredom proneness would serve as sequential indirect roles in the relationship between problematic short-form video use and appearance anxiety.

## Methods

### Participants and procedure

The study recruited a total of 411 college students from China. The sample had a mean age of 21.52 years (*SD* = 2.30), with females comprising 62.5% of the participants. The demographic composition included freshmen (8.8%), sophomores (13.4%), juniors (18.5%), seniors (51.8%), and postgraduate students (7.5%). Slightly over half of the participants (51.1%) majored in natural sciences, and the majority (71.5%) reported residing in urban areas.

This study received ethical approval from the University’s Research Ethics Committee and was conducted in accordance with the principles of the Declaration of Helsinki. Data were collected using an online questionnaire administered via the WENJUANXING platform. Online informed consent was obtained from all participants prior to their involvement. Participants were advised that their participation was voluntary and that they could withdraw from the study at any time without penalty.

## Measures

### Problematic short-form video use

Problematic short-form video use was measured using the Chinese version of Facebook Intrusion Questionnaire [48]. To capture behaviors specific to short-form video consumption, we replaced the term “Facebook” with “short-form video app” in all items. Sample items: “I often use short-form video app for no particular reason” and “I have been unable to reduce my short-form video app use”. The scale consists of eight items rated on a 5-point Likert scale from 1 (“not at all true”) to 5 (“very true”), with higher scores reflecting greater problematic short-form video use. The scale demonstrated good internal consistency in Chinese college students (α = 0.89; [49]) and in the present study (α = 0.88).

### Appearance anxiety

Appearance anxiety was assessed using the Chinese version of Social Appearance Anxiety Scale [2]. The scale comprises 16 items rated on a 5-point Likert scale ranging from 1 (“not at all true”) to 5 (“very true”). One item (e.g., “I feel comfortable with the way I appear to others”) was reverse-coded prior to analysis. Higher total scores reflect greater levels of appearance anxiety. The scale demonstrated excellent internal consistency in Chinese college students (α = 0.94; [50]) and in the present study (α = 0.95).

### Self-esteem

Self-esteem was measured using the Chinese version of Rosenberg Self-Esteem Scale [22]. The instrument consists of ten items (e.g., “On the whole, I am satisfied with myself”) rated on a 4-point Likert scale from 1 (strongly disagree) to 4 (strongly agree), with higher total scores indicating more positive self-esteem. The scale demonstrated good internal consistency in Chinese college students (α = 0.87; [50]) and in the present study (α = 0.86).

### Boredom proneness

Boredom proneness was measured using the Chinese version of Boredom Proneness Scale [36]. Sample items include “It is easy for me to concentrate on my activities” and “I find it easy to entertain myself.” The scale rated on a 7-point Likert scale ranging from 1 (highly disagree) to 7 (highly agree), with higher total scores indicating a greater tendency toward boredom proneness. The scale showed acceptable internal consistency in Chinese students (α = 0.89; [51]) and in the present study (α = 0.73).

### Data analysis

Descriptive statistics and Pearson correlation analyses were performed using SPSS (version 27.0). All variables were standardized before proceeding with further analyses. The mediating effects were tested using the PROCESS macro (version 4.0). Significance of the indirect effects was assessed using bias-corrected bootstrap confidence intervals (CI) derived from 5,000 resamples. An effect was considered statistically significant if the 95% confidence interval did not include zero. To interpret the magnitude of the correlation coefficients, we used the benchmarks of |*r*| = 0.10 (small), 0.20 (medium), and 0.30 (large) [52]. We also followed Keith’s benchmarks for standardized regression coefficients, with |β| ≥ 0.05 (small), ≥ 0.10 (moderate), and ≥ 0.25 (large) [53].

## Results

### Preliminary analyses

Descriptive statistics, including means, standard deviations, skewness, and kurtosis, along with bivariate correlations among the study variables, were presented in Table 1. The values of skewness and kurtosis indicated that all variables approximated a normal distribution, in accordance with conventional thresholds (i.e., absolute skewness < 2.0 and absolute kurtosis < 7.0; [54]). As expected, problematic short-form video use showed large positive associations with boredom proneness (*r* = 0.34, *p* < 0.01) and appearance anxiety (*r* = 0.51, *p* < 0.01), and a medium negative association with self-esteem (*r* = −0.26, *p* < 0.01). Self-esteem demonstrated strong negative correlations with both boredom proneness (*r* = −0 .63, *p* < 0.01) and appearance anxiety (*r* = −0 .50, *p* < 0.01). Boredom proneness also showed a large positive correlation with appearance anxiety (*r* = 0.45, *p* < 0 .01).

**Table 1:** Descriptive statistics and correlations among the study variables (*N* = 411)

Variables	M	SD	1	2	3	4
1. Problematic short-form video use	21.19	6.98	—			
2. Self-esteem	29.46	4.84	−0.26^**^	—		
3. Boredom proneness	43.41	8.91	0.34^**^	−0.63^**^	–	
4. Appearance anxiety	47.92	14.03	0.51^**^	−0.50^**^	0.45^**^	–
Skewness	–	–	0.16	0.21	−0.77	−0.25
Kurtosis	–	–	−0.70	−0.50	0.46	−0.88

^**^p < 0.01

### The indirect effect model analyses

The results of the indirect effect model were summarized in Table 2. Problematic short-form video use exhibited a large positive association with appearance anxiety (β = 0.38, *p* < 0.001) and a moderate positive association with boredom proneness (β = 0.20, *p* < 0.001), and a large negative association with self-esteem (β = −0.26, *p* < 0.001). Self-esteem showed strong negative associations with boredom proneness (β = −0.57, *p* < 0.001) and appearance anxiety (β = −0.33, *p* < 0.001). Boredom proneness was positively, albeit more modestly, associated with appearance anxiety (β = 0.12, *p* < 0.05).

Bootstrap analyses (based on 5,000 resamples) indicated that all indirect effects were different from zero (95% CIs excluded zero; Table 2). The indirect pathway via self-esteem (PSVU → SE → AA; β = 0.12, 95% CI [0.08, 0.18]) was numerically larger than the pathways via boredom proneness alone (PSVU → BP → AA; β = 0.02, 95% CI [0.002, 0.05]) and the serial pathway (PSVU → SE → BP → AA; β = 0.02, 95% CI [0.002, 0.04]). Overall, these results suggest that self-esteem and boredom proneness function as independent and sequential indirect factors in the relationship between problematic short-form video use and appearance anxiety. Specifically, higher levels of problematic short-form video use were associated with lower self-esteem and greater boredom proneness, which were in turn linked to elevated appearance anxiety.

**Table 2:** The results of the indirect effect model (*N* = 411)

Independent variable	Dependent variable	β	SE	t
PSVU	SE	−0.26	0.05	−5.41^***^
Gender		0.02	0.10	0.21
PSVU	BP	0.20	0.04	5.03^***^
SE		−0.57	0.04	−14.80^***^
Gender		0.03	0.08	0.41
PSVU	AA	0.38	0.04	9.39^***^
SE		−0.33	0.05	−6.67^***^
BP		0.12	0.05	2.31^*^
Gender		0.15	0.08	1.91
Indirect effects	β	SE	95% LLCI	95% ULCI
PSVU → SE → AA	0.12	0.02	0.08	0.18
PSVU → BP → AA	0.02	0.01	0.002	0.05
PSVU → SE → BP → AA	0.02	0.009	0.002	0.04

PSVU = problematic short-form video use, SE = self-esteem, BP = boredom proneness, AA = appearance anxiety, ^*^p < 0.05, ^***^p < 0.001

## Discussion

This study examined the relationship between problematic short-form video use and appearance anxiety, with a specific focus on the indirect roles of self-esteem and boredom proneness. Overall, problematic short-form video use showed a strong positive association with appearance anxiety and was also linked to appearance anxiety indirectly through self-esteem and boredom proneness, both independently and sequentially. These findings provide theoretical and practical insights into psychological factors that may help explain the association between problematic short-form video use and appearance anxiety among young adults.

### Problematic short-form video use and appearance anxiety

A large positive association was observed between problematic short-form video use and appearance anxiety, confirming Hypothesis 1 and aligning with recent empirical findings [10, 17]. Although the present study did not directly measure appearance-based social comparison, the observed association is consistent with prior theorizing and research suggesting that short-form video platforms frequently expose users to highly curated and idealized appearance-related content, which may increase the salience of appearance-related evaluation and comparison opportunities. Such processes have been linked to body dissatisfaction and appearance-related distress in previous work [55]. In a similar vein, objectification-based perspectives propose that visually oriented media environments may encourage greater appearance monitoring and self-objectification, which have been associated with higher appearance anxiety [56, 57]. Importantly, these mechanisms are offered as plausible interpretations rather than conclusions that can be directly inferred from the current data.

It is also noteworthy that young women constituted more than half of the sample (62.5%). Although prior research suggests that young women may be more likely to engage with beauty- and fashion-oriented content and may report greater appearance-related concerns [7], gender was included as a covariate in our indirect model and was not a significant predictor in the relevant equations (Table 2). This suggests that the observed associations were not simply attributable to gender differences in this sample; nevertheless, future research could further examine potential gender-related heterogeneity. Interventions that strengthen media literacy has shown promise in reducing appearance-related distress and may be relevant to prevention efforts in short-form video contexts [58].

### Indirect effects of self-esteem and boredom proneness

The analysis revealed that self-esteem served as an indirect role in the relationship between problematic short-form video use and appearance anxiety, supporting Hypothesis 2. This finding aligns with previous studies linking problematic social media use to reduced self-esteem [29–31]. Diminished self-esteem, in turn, represents a well-established risk factor for appearance anxiety, as individuals with lower self-worth demonstrate greater susceptibility to negative body image and heightened fear of social evaluation [59, 60]. These results are broadly in line with self-discrepancy theory [61], which proposes that perceived discrepancies between one’s actual and ideal selves are associated with lower self-esteem and greater anxiety. The findings are also consistent with objectification theory [16], which posits that appearance-salient media contexts can encourage self-objectification by repeatedly directing attention to how one looks from an observer’s perspective. In short-form video environments, beauty filters and appearance-enhancing tools may heighten attention to one’s looks and encourage evaluating the self from an observer’s perspective, while algorithmic recommendation systems may repeatedly prioritize and amplify highly curated, appearance-focused content, thereby sustaining exposure to social-evaluative cues. These platform features may foster self-objectification and appearance monitoring, which have been linked to lower self-worth and greater appearance-related distress. Notably, self-discrepancy and self-objectification were not directly measured in this study; thus, these frameworks should be viewed as interpretive lenses that motivate further research rather than as empirically tested mechanisms in the present dataset.

Boredom proneness also demonstrated an indirect role in the relationship between problematic short-form video use and appearance anxiety, confirming Hypothesis 3. This result is consistent with longitudinal evidence indicating that mobile phone addiction was related to increased boredom proneness [62], which in turn is strongly linked to elevated appearance anxiety. It is plausible that repeated engagement with rapidly rewarding short-form content may relate to reduced tolerance for low-stimulation contexts or a stronger preference for novelty, which could be associated with higher boredom proneness [63]. Moreover, individuals higher in boredom proneness may be more likely to seek stimulation and may engage more frequently with appearance-salient content; when exposed to idealized representations of beauty or physical fitness, upward appearance-related comparisons may occur. Such comparisons have been associated with body dissatisfaction and negative self-evaluations and may relate to higher appearance anxiety [55, 64]. Importantly, appearance-based social comparison was not directly measured and should be explicitly assessed in future studies to test this proposed pathway.

Finally, the analysis demonstrated a serial indirect effect through self-esteem and boredom proneness, collectively accounting for a substantial portion of the relationship between problematic short-form video use and appearance anxiety, thereby supporting Hypothesis 4. These findings indicate that problematic short-form video use was associated with appearance anxiety both directly and indirectly, through the pathways of lower self-esteem and higher boredom proneness. The results highlight the interplay between platform-specific environmental factors and individual psychological vulnerabilities in the development of appearance anxiety. From a practical perspective, these results tentatively point to the potential value of interventions that bolster self-esteem and promote engagement in meaningful and goal-directed activities. However, intervention implications should be evaluated in longitudinal and experimental research that can more clearly establish temporal ordering and causal effects.

### Limitations and implications

This study is subject to several limitations. Firstly, reliance on self-reported data may introduce response biases such as social desirability or recall inaccuracies. Future research could incorporate multi-method assessments, such as peer evaluations (e.g., close friends’ reports of appearance-related reassurance seeking and appearance-focused talk) or/and behavioral data (e.g., time spent on short-form video apps and engagement with appearance-salient content), to enhance the validity of the measurements. Secondly, the cross-sectional design precludes definitive causal conclusions. Longitudinal or experimental studies are warranted to establish temporal precedence and clarify directional relationships among the variables. Thirdly, while the current model focused on intrapersonal factors such as self-esteem and boredom proneness, it did not account for potentially influential environmental and social factors, including social support [65] and peer relationship quality [66]. Incorporating these contextual and relational dimensions in future studies could provide a more comprehensive understanding of the factors associated with appearance anxiety. Finally, the measures were originally developed in Western contexts, and our sample consisted of Chinese young adults. Although validated Chinese versions of these measures have been used and supported in prior research, the constructs may still be influenced by cultural context. For example, in collectivistic cultures (e.g., China), appearance anxiety may be shaped by culturally specific beauty ideals and social comparison norms, which could affect both its level and its associations with other study variables. Thus, caution is warranted when generalizing our findings beyond Chinese young adults. Future research could benefit from testing measurement invariance across cultures to clarify how these constructs function in different cultural settings.

Notwithstanding these limitations, the present study provides valuable insights for addressing appearance anxiety among young adults in an increasingly digitalized society. Given the central role of self-esteem in the observed indirect pathways, preventive and therapeutic strategies targeting self-enhancement may help reduce negative body image outcomes. Structured interventions designed to bolster self-esteem [67] may serve as an effective means to counteract the adverse psychological effects associated with problematic short-form video use. Meta-analytic evidence supports the efficacy of certain psychosocial approaches, such as cognitive behavioral therapy and reminiscence-based interventions, in promoting self-esteem [68, 69], suggesting their potential relevance in this context. Beyond its relevance to the general young adult population, these findings may also hold important implications for individuals with or at risk of eating disorders. Appearance anxiety and low self-esteem are well-documented risk factors for disordered eating behaviors [5, 67]. While the present study did not assess disordered eating directly, problematic short-form video use could plausibly co-occur with these vulnerabilities by increasing exposure to idealized body images and appearance-salient cues [6]. Future research should directly examine disordered eating outcomes and test whether the pathways identified here generalize to eating-related risk.

## Conclusion

The present study underscores a large positive association between problematic short-form video use and appearance anxiety among young adults, with self-esteem and boredom proneness serving as indirect factors both independently and sequentially. These findings contribute to the existing literature by elucidating psychological pathways through which problematic short-form video use may be linked to appearance anxiety, thereby enriching theoretical frameworks within digital media effects and body image research. The results imply that effective interventions to reduce appearance anxiety should consider not only excessive short video consumption but also its associated psychological factors, particularly low self-esteem. Future studies would benefit from longitudinal or experimental designs, direct assessment of proposed mechanisms (e.g., appearance-based social comparison, self-objectification, and self-discrepancy), and culturally informed approaches to strengthen generalizability and contextual relevance.

## Data Availability

The data are available from the first author upon reasonable request.
